# Association of Social and Economic Inequality With Coronavirus Disease 2019 Incidence and Mortality Across US Counties

**DOI:** 10.1001/jamanetworkopen.2020.34578

**Published:** 2021-01-20

**Authors:** Tim F. Liao, Fernando De Maio

**Affiliations:** 1 Department of Sociology, University of Illinois at Urbana-Champaign; 2 Center for Health Equity, American Medical Association, Chicago, Illinois; 3 Department of Sociology, DePaul University, Chicago, Illinois

## Abstract

**Importance:**

It is now established that across the United States, minoritized populations have borne a disproportionate burden from coronavirus disease 2019 (COVID-19). However, little is known about the interaction among a county’s racial/ethnic composition, its level of income inequality, political factors, and COVID-19 outcomes in the population.

**Objective:**

To quantify the association of economic inequality, racial/ethnic composition, political factors, and state health care policy with the incidence and mortality burden associated with COVID-19.

**Design, Setting, and Participants:**

This cross-sectional study used data from the 3142 counties in the 50 US states and for Washington, DC. Data on the first 200 days of the COVID-19 pandemic, from the first confirmed US case on January 22 to August 8, 2020, were gathered from the Centers for Disease Control and Prevention and USAFacts.org, the US Census Bureau, the American Community Survey, GitHub, the Kaiser Family Foundation, the Council of State Governments, and the National Governors Association.

**Exposures:**

Racial/ethnic composition was determined as percentage of the population that is Black or Hispanic; income inequality, using the Gini index; politics, political affiliation and sex of the state governor, gubernatorial term limits, and percentage of the county’s population that voted Republican in 2016; and state health care policy, participation in the expansion of Medicaid under the Affordable Care Act. Six additional covariates were assessed.

**Main Outcomes and Measures:**

Cumulative COVID-19 incidence and mortality rates for US counties during the first 200 days of the pandemic. Main measures include percentage Black and Hispanic population composition, income inequality, and a set of additional covariates.

**Results:**

This study included 3141 of 3142 US counties. The mean Black population was 9.365% (range, 0-86.593%); the mean Hispanic population was 9.754% (range, 0.648%-96.353%); the mean Gini ratio was 44.538 (range, 25.670-66.470); the proportion of counties within states that implemented Medicaid expansion was 0.577 (range, 0-1); the mean number of confirmed COVID-19 cases per 100 000 population was 1093.882 (range, 0-14 019.852); and the mean number of COVID-19–related deaths per 100 000 population was 26.173 (range, 0-413.858). A 1.0% increase in a county’s income inequality corresponded to an adjusted risk ratio (RR) of 1.020 (95% CI, 1.012-1.027) for COVID-19 incidence and adjusted RR of 1.030 (95% CI, 1.012-1.047) for COVID-19 mortality. Inequality compounded the association of racial/ethnic composition through interaction, with higher income inequality raising the intercepts of the incidence curve RR by a factor of 1.041 (95% CI, 1.031-1.051) and that of the mortality curve RR by a factor of 1.068 (95% CI, 1.042-1.094) but slightly lowering their curvatures, especially for Hispanic composition. When state-level specificities were controlled, none of the state political factors were associated with COVID-19 incidence or mortality. However, a county in a state with Medicaid expansion implemented would see the incidence rate RR decreased by a multiplicative factor of 0.678 (95% CI, 0.501-0.918).

**Conclusions and Relevance:**

This county-level ecological analysis suggests that COVID-19 surveillance systems should account for county-level income inequality to better understand the social patterning of COVID-19 incidence and mortality. High levels of income inequality may harm population health irrespective of racial/ethnic composition.

## Key Points

**Question:**

Are racial/ethnic population composition and economic inequality associated with coronavirus disease 2019 (COVID-19) incidence and mortality?

**Findings:**

This cross-sectional ecological analysis of cumulative COVID-19 incidence and mortality rates for the first 200 days of the pandemic in 3141 US counties confirmed positive associations of incidence and mortality rates with racial/ethnic composition and with income inequality well as a joint association of incidence and mortality with both structural factors.

**Meaning:**

This study suggests that COVID-19 surveillance systems should take into account county-level income inequality to better understand the social patterning of COVID-19.

## Teaser

This cross-sectional ecological analysis quantifies the association of economic inequality, racial/ethnic composition, political factors, and state health care policy with the incidence and mortality burden associated with coronavirus disease 2019 (COVID-19) at the US county level.

## Introduction

The disproportionate burden of coronavirus disease 2019 (COVID-19) on minoritized populations in the United States is now well established.^1,2^ The latest data indicate that age-adjusted mortality rates among Black and Hispanic populations are 3.6 and 3.2 times higher, respectively, than for the non-Hispanic White population.^3^ This difference translates into almost 30 000 excess deaths among Black and Hispanic residents in the United States,^3^ a figure expected to increase substantially in the coming months, even under conservative modeling assumptions.

Understanding inequities in the context of COVID-19 requires not just a descriptive analysis of racial/ethnic gaps but also explanatory studies that identify the structural drivers of those gaps.^4^ Such work is vitally important for shifting the narrative from one that places explanatory power on race to one that recognizes the centrality of racism in shaping health.^5^ Based on previous research in social epidemiology,^6^ there is reason to expect that structural drivers shaping health inequities may be reflected in a community’s level of income inequality. As an upstream determinant of health, income inequality influences both community-level characteristics (including the quality and accessibility of housing, education, transportation, and other aspects of public infrastructure) and individual-level physiological processes (primarily through the activation of cortisol and other stress responses).^7^ However, little is known about the role of income inequality as a driver of racial/ethnic inequities with COVID-19.

Along with income inequality, the structural drivers of health inequities may also be reflected by political attributes of places, signaling the increasing recognition of political determinants of health.^8^ A growing literature in the social sciences explores the link between right-wing policies and population health,^9^ and COVID-19 studies^10,11,12^ have found associations between state-level political characteristics such as a governor’s party affiliation, sex, and term limit—as well as between a county’s proportion of Republican votes in the 2016 presidential election—and overall incidence and mortality outcomes. Governors facing a binding term limit may exhibit different political behavior from those able to run again.^13^

Although researchers have examined the association between COVID-19 outcomes and racial/ethnic composition in either a simple correlation analysis at the county level or a linear regression analysis at the individual level without focusing on incidence/mortality,^14,15^ no study, to our knowledge, has examined the joint associations of income inequality, racial/ethnic composition, and political attributes of places with COVID-19 outcomes. The objective of this investigation is to analyze the US county-level associations of income inequality, racial/ethnic composition, and political attributes with COVID-19 incidence and mortality in the first 200 days of the pandemic, covering the first 2 major peaks in daily case counts in the country.^16^

## Methods

### Data

This cross-sectional study used data from the 3142 counties in the 50 states and for Washington, DC. The 7 major sources included the Centers for Disease Control and Prevention and USAFacts.org,^17^ the US Census Bureau,^18^ the American Community Survey,^19^ GitHub,^20^ the Kaiser Family Foundation,^21^ the Council of State Governments,^22^ and the National Governors Association^23^ for the outcome and the covariate variables. Table 1 presents a description of the data, including the variable names, definitions, means and ranges of the variables, and their sources. The analysis included 3141 of 3142 counties, with Rio Arriba County, New Mexico, excluded because of missing information on income inequality. The data are for the first 200 days of the pandemic from the first confirmed US case on January 22 to August 8, 2020.^16^ This study represents, to our knowledge, the first attempt to combine these data sources in a single analysis not reported or considered in other places. Because all of the data used in the study are at the county or state level and in the public domain, the study was exempted by the University of Illinois institutional review board. This study followed the Strengthening the Reporting of Observational Studies in Epidemiology (STROBE) reporting guideline.

**Table 1.** Descriptions of the Variables in the Analysis of 3141 US Counties

Variable	Definition	Mean (range)	Source
Incidence	No. of confirmed cases per 100 000 population	1093.882 (0-14 019.852)	CDC and USAFacts.org; US Census Bureau
Mortality	No. of deaths per 100 000 population	26.173 (0-413.858)	CDC and USAFacts.org; US Census Bureau
Male	Male population, 2019, %	50.116 (42.992-73.486)	US Census Bureau
Aged <20 y	Population aged <20 y, 2019, %	12.201 (0-22.443)	US Census Bureau
Aged ≥70 y	Population aged ≥70 y, 2019, %	6.751 (1.597-21.939)	US Census Bureau
ACA	States implemented Medicaid expansion, 2020, proportion	0.577 (0-1)	kff.org, August 2020
Time since first case	No. of days	128.173 (0-200)	CDC and USAFacts.org
Population density	Population per square kilometer, 2019	151.458 (1.3-11 569.7)	US Census Bureau
Black	Black population, 2019, %	9.365 (0-86.593)	US Census Bureau
Hispanic	Hispanic population, 2019, %	9.754 (0.648-96.353)	US Census Bureau
Gini index for income inequality	0 Indicates perfect equality and 100, perfect inequality	44.538 (25.670-66.470)	2018 ACS
Governor term limit	1 Indicates yes	0.182 (0-1)	Council of State Governments
Governor party	1 Indicates Republican	0.569 (0-1)	National Governors Association
Governor sex	1 Indicates male	0.838 (0-1)	National Governors Association
Republican vote	2016 Republican vote in a county, %	63.508 (4.122-95.273)	GitHub with 3 county-specific additions

Abbreviations: ACA, Affordable Care Act; ACS, American Community Survey; CDC, Centers for Disease Control and Prevention.

### Measures

The numbers of cumulative confirmed cases and deaths in a county (both per 100 000 population) are the 2 outcome variables. To estimate the association of incidence and mortality with racial/ethnic composition, economic inequality, and political characteristics, we controlled for 6 potential confounding factors. These factors include percentages of population that were male, younger than 20 years, and 70 years or older; population density per square kilometer; number of days since the first confirmed local case; and county population size (used to compute incidence and mortality rates). The analysis included 3 relevant state-level political variables—whether a governor faced a term limit, was Republican, or was male—as well as whether a state implemented Medicaid expansion under the Affordable Care Act (ACA).

The analysis included 3 key structural variables. First, to explore patterns by racial/ethnic composition, the analysis used percentages of Black and Hispanic populations. Second, to measure income inequality, the analysis used the Gini index. Last, to explore the political associations of health, the analysis included 3 relevant state-level political variables described above and a county-level political variable recording the percentage of the county’s population that voted Republican in the 2016 presidential election. Finally, the analysis included whether a state implemented ACA Medicaid expansion for assessing a state’s health care type.

### Statistical Analysis

Both outcomes are heavily skewed to the right and can be described by a negative binomial distribution. To model such outcomes, multilevel negative binomial regressions with level-2 (counties nested in states) random intercepts and a log link were applied. The log link implied that exponentiated coefficient estimates provide relative risks (or risk ratios [RRs]) of incidence and mortality for ease of interpretation. Two-sided *P* < .05 indicated statistical significance. Analyses were performed using STATA, version 16.0 (StataCorp LLC).

## Results

### Main Findings

This study analyzed the association between COVID-19 incidence and mortality rates of 3141 US counties and the following: the percentage of Black and Hispanic populations; the Gini index for income inequality; a governor’s sex, political affiliation, and term limit; percentage of population voting Republican in 2016 in a county; a state’s health care system type; and a set of control variables. Within the 3141 analyzed counties, the mean Black population was 9.365% (range, 0-86.593%); the mean Hispanic population was 9.754% (range, 0.648%-96.353%); the mean Gini ratio was 44.538 (range, 25.670-66.470); the proportion of counties within states that implemented Medicaid expansion was 0.577 (range, 0-1); the mean number of confirmed COVID-19 cases per 100 000 population was 1093.882 (range, 0-14 019.852); and the mean number of COVID-19–related deaths per 100 000 population was 26.173 (range, 0-413.858). Table 2 presents estimated RRs for incidence and mortality based on the data from the first 200 days of the pandemic in the United States. With controlling for other variables, a 1.0% increase in a county’s Black population corresponded to a 1.9% increase in incidence (RR, 1.019; 95% CI, 1.016-1.022) and 2.6% increase in mortality (RR, 1.026; 95% CI, 1.020-1.033; models 1 and 3). A 1.0% increase in a county’s Hispanic population corresponded to a 2.4% increase in incidence (RR, 1.024; 95% CI, 1.012-1025) and a 1.9% increase in mortality (RR, 1.019; 95% CI, 1.012-1.025; models 1 and 3). A 1.0% rise in a county’s income inequality corresponded to a 2.0% rise in incidence (RR, 1.020; 95% CI, 1.012-1.027) and 3.0% rise in mortality (RR, 1.030; 95% CI, 1.012-1.047; models 1 and 3). None of the state-level political characteristics—Republican governor, male governor, or governor facing a term limit—were associated with COVID-19 incidence or mortality in the multilevel analysis. However, an association was discovered for ACA Medicaid expansion: a county in a state with ACA Medicaid expansion had a mean 32% lower incidence rate compared with counties in states that did not participate in ACA expansion (models 1 and 2). Participation in ACA expansion was not associated with mortality rates (models 3 and 4).

**Table 2.** Estimated Incidence and Mortality RR From Multilevel Negative Binomial Models of Incidence and Mortality in 3141 US Counties

County-level covariate	Incidence rate				Mortality rate			
	Model 1		Model 2		Model 3		Model 4	
	RR (95% CI)	*P* value	RR (95% CI)	*P* value	RR (95% CI)	*P* value	RR (95% CI)	*P* value
Male population	1.034 (1.021-1.048)	<.001	1.036 (1.022-1.049)	<.001	1.007 (0.978-1.038)	.63	1.008 (0.978-1.038)	.06
Population aged <20 y	2713.809 (243.927-30 192.525)	<.001	2111.216 (191.856-23 232.120)	<.001	2 983 154.061 (9754.23-9.123 × 10^8^)	<.001	1 275 284.454 (4373.45-3.719 × 10^8^)	<.001
Population aged ≥70 y	0.220 (0.020-2.386)	.21	0.342 (0.032-3.687)	.38	19 579.380 (64.410-5951 385.453)	.001	18 909.690 (63.609-5 621 431.000)	.001
Time since first case	1.010 (1.009-1.011)	<.001	1.010 (1.009-1.011)	<.001	1.014 (1.012-1.017)	<.001	1.015 (1.013-1.017)	<.001
Population density	7.232 × 10^16^ (0.387-1.353 × 10^34^)	.06	8.414 × 10^20^ (9154.060-7.733 × 10^37^)	.02	5.403 × 10^41^ (0.000-1.445 × 10^87^)	.07	1.153 × 10^46^ (92.082-1.444 × 10^90^)	.04
ACA Medicaid expansion	0.678 (0.501-0.918)	.01	0.681 (0.503-0.921)	.01	1.080 (0.681-1.714)	.74	1.105 (0.700-1.745)	.67
Governor term Limit	1.217 (0.886-1.672)	.23	1.201 (0.874-1.651)	.26	1.193 (0.732-1.944)	.48	1.174 (0.724-1.903)	.52
Governor Republican	1.097 (0.845-1.423)	.49	1.103 (0.850-1.433)	.46	0.873 (0.587-1.300)	.51	0.881 (0.594-1.306)	.53
Governor male	1.116 (0.814-1.529)	.50	1.109 (0.809-1.521)	.52	1.132 (0.699-1.833)	.61	1.115 (0.692-1.794)	.66
Republican vote	1.003 (1.000-1.005)	.046	1.002 (1.000-1.005)	.11	0.999 (0.993-1.005)	.79	1.000 (0.994-1.006)	.97
Black population	1.019 (1.016-1.022)	<.001	1.042 (1.023-1.063)	<.001	1.026 (1.020-1.033)	<.001	1.076 (1.029-1.125)	.001
Hispanic population	1.024 (1.021-1.027)	<.001	1.090 (1.069-1.112)	<.001	1.019 (1.012-1.025)	<.001	1.105 (1.062-1.149)	<.001
Gini index	1.020 (1.012-1.027)	<.001	1.041 (1.031-1.051)	<.001	1.030 (1.012-1.047)	.001	1.068 (1.042-1.094)	<.001
Black population × Gini index	NA	NA	0.999 (0.999-1.000)	.01	NA	NA	0.999 (0.998-1.000)	.03
Hispanic population × Gini index	NA	NA	0.999 (0.998-0.999)	<.001	NA	NA	0.998 (0.997-0.999)	<.001
Additional state covariates	0.375 (0.358-0.394)	.000	0.371 (0.353-0.389)	<.001	1.839 (1.739-1.944)	<.001	1.826 (1.727-1.932)	<.001
Model χ^2^ value (*df*)	1762.98 (13)		1826.95 (15)		439.58 (13)		471.80 (15)	

Abbreviations: ACA, Affordable Care Act; NA, not applicable.

### Interactions Between Racial/Ethnic Composition and Income Inequality

**Figure 1.**
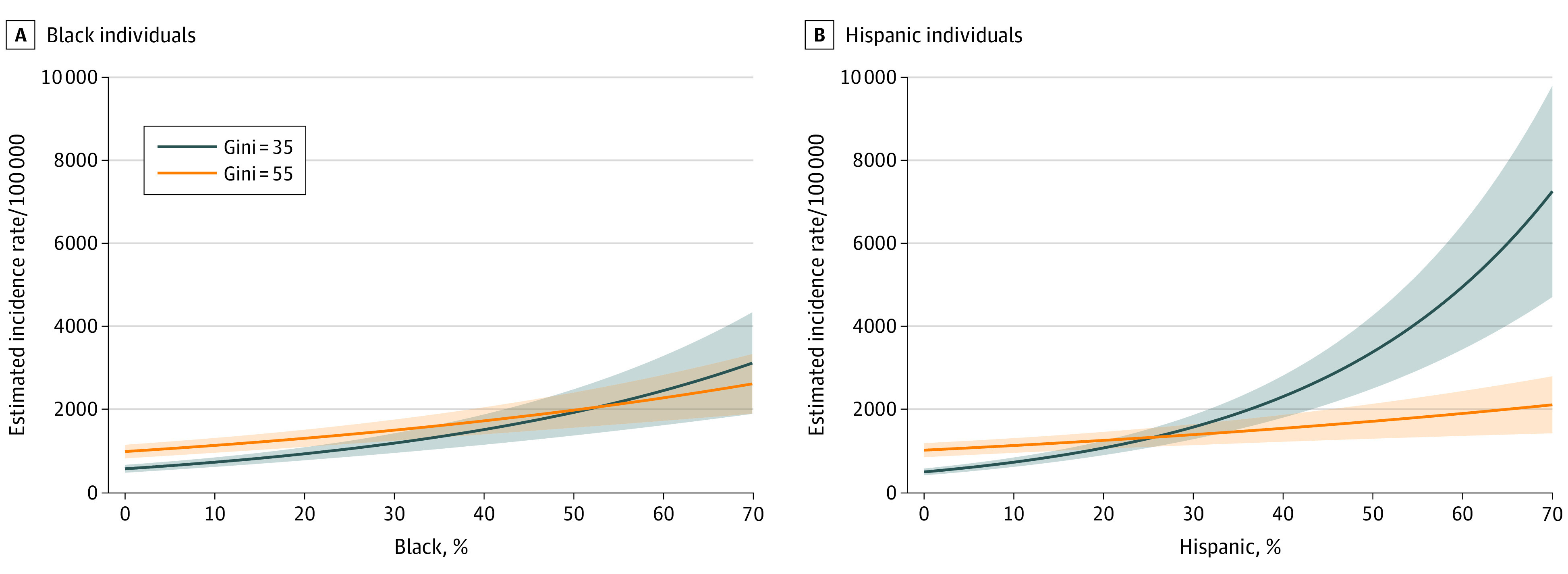
Incidence Estimations by County-Level Racial/Ethnic Composition and Gini Index for Income Inequality The Gini index ranges from 0, indicating perfect equality, to 100, indicating perfect inequality.

An interaction was found between racial/ethnic composition and income inequality. Figure 1 plots estimated COVID-19 confirmed infection rates by racial/ethnic composition and income inequality, including their interaction, with controlling for all other covariates. The computation was based on the observed values of these key structural variables within most of the counties, with all other variables set at their mean values. Three patterns emerge. First, COVID-19 incidence rates were higher among counties for each percentage increase of Black residents and especially of Hispanic residents (RR, 1.042; 95% CI, 1.042-1.063). Second, income inequality interacted with the association between incidence and racial/ethnic composition, with higher income inequality not only raising the intercepts but also lowering the slopes of the incidence curves, more so for Hispanic than for Black composition (RR, 0.999 [95% CI, 0.999-1.000] for Black vs 0.999 [95% CI, 0.998-0.999] for Hispanic composition), indicating that the interaction between Black composition and income inequality was not as strong as that between Hispanic composition and income equality. Finally, income inequality was most strongly associated with higher COVID-19 incidence in counties with relatively low proportions of Black or Hispanic residents, indicated by the greater separation of the 2 curves toward the lower end of Figure 1 (estimated incidence rate [IR] for Black composition, 587.26 [95% CI, 493.41-681.12] at Gini value of 35% and 1001.69 [95% CI, 838.91-1164.48] at Gini value of 55%; for Hispanic composition: IR, 505.96 [95% CI, 425.64-588.28] at Gini value of 35% and 1918.08 [95% CI, 1535.14-2301.02] at Gini value of 55%).

**Figure 2.**
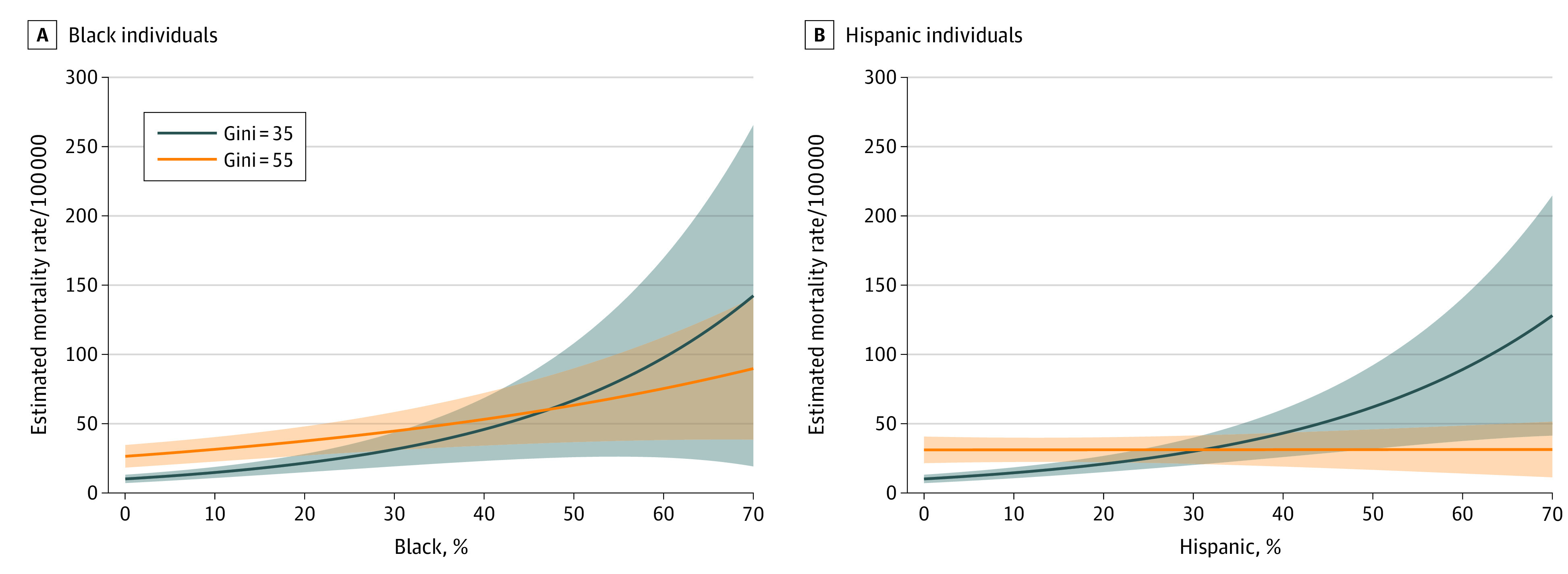
Mortality Predictions by County-Level Racial/Ethnic Composition and Gini Index for Income Inequality The Gini index ranges from 0, indicating perfect equality, to 100, indicating perfect inequality.

In a similar presentation, Figure 2 plots estimated COVID-19 mortality rates by racial/ethnic composition and income inequality with their interaction by controlling other covariates. The graph presents similar findings to those in Figure 1, especially in terms of the separation between the 2 mortality curves at the low and high levels of inequality (estimated mortality rate [MR] for Black composition, 10.13 [95% CI, 7.14-13.13] at Gini value of 35% and 26.44 [95% CI, 18.29-34.59] at Gini value of 55%; for Hispanics, 10.13 [95% CI, 7.11-13.16] at Gini value of 35% and 36.02 [95% CI, 23.00-49.04] at Gini value of 55%). Income inequality interacted with the association between mortality and racial/ethnic composition, with higher income inequality raising the intercepts (RR, 1.068; 95% CI, 1.042-1.094) but lowering the slopes of the mortality curves more so for Hispanic composition (RR, 0.998; 95% CI, 0.997-0.999) than for Black composition (RR, 0.999; 95% CI, 0.999-1.000). There were 2 exception to the similarities. First, for income inequality levels at or below Gini values of 35%, there were no counties with Black composition greater than 5% and no counties with Hispanic composition greater than 50%. Second, among those counties with inequality values at or below 35%, no counties reported any mortality, although a few reported incidence. Hence, there was much a wider 95% CI for the estimated Black mortality rate than the counterpart 95% CI for the estimated Hispanic mortality rate, and the 95% CIs for the lower inequality level for either racial composition were not narrower than those for the higher inequality level. Finally, when income inequality was high, the association between estimated mortality rate and Black composition was still positive, but it was almost absent between estimated mortality rate and Hispanic composition.

## Discussion

Many studies have concluded that COVID-19 has revealed the fault lines of inequality in the United States. Already, published research has shown a disproportionate burden of COVID-19 on minoritized populations.^1,2^ This study expands that picture by illustrating how county-level income inequality matters, in itself and through its interaction with racial/ethnic composition to systematically disadvantage Black and Hispanic communities. For the nation to gain control of COVID-19 incidence and mortality, it is essential that public health and government officials know which places are most affected. This analysis found racial/ethnic composition, while important, does not reveal the full complexity of the story. Income inequality—a measure not typically included in public health county-level surveillance—also needs to be considered as a driver of the disproportionate burden borne by minoritized communities across the United States.

Past studies that did not rely on multilevel analysis showed the association between state-level political factors and COVID-19 outcomes.^10,11,12^ In contrast to their findings, the present study did not substantiate such political influence once states’ specificities were modeled with a multilevel data analysis framework. This analytic framework, however, did not erase all state-level influences. Counties in states that participated in ACA Medicaid expansion were found to have a sizable reduction in incidence rates (models 1 and 2).

This study used county-level population data of the first 200 days of the pandemic in the United States to add to the growing literature documenting the harmful effects of racialized economic inequality.^6^ It must be emphasized that these processes are social rather than biological or genetic.^24^ The analyses point to the fundamental cause of racialized economic inequality, not race, as a risk factor producing health inequities. It also points to the complex association between racial/ethnic composition and COVID-19 incidence and mortality, conditional on income inequality. Across all counties, a higher Black or Hispanic population composition was always associated with a higher incidence and a higher mortality. However, at lower levels of inequality, the association between racial/ethnic composition and COVID-19 outcomes was greater than when inequality levels were higher. High levels of income inequality harm population health (indicated by high levels of COVID-19 incidence and mortality), irrespective of racial/ethnic composition. This may reflect what income inequality researchers have called the *neomaterial pathway*, linking high income inequality to the breakdown of critical social systems and public infrastructure, including education, transportation, and health care.^25,26^ At lower levels of Black or Hispanic racial/ethnic composition, this study found that the burden of COVID-19 was more segmented by income inequality. This finding implies that counties with relatively low proportions of Black or Hispanic residents may experience health effects of income inequality associated with the neomaterial pathway, which connects income inequality to population health through the breakdown of public infrastructure. On the other hand, higher levels of Black and Hispanic composition often coincide with places of higher income inequality, and because both structural factors are at a high level, a further increase of Black or Hispanic residents in the racial/ethnic composition was no longer associated with additional health burden. This situation is consistent with the psychosocial pathway, a central plank in the income inequality and health literature. That body of literature rests on stress pathways to model how inequality gets under the skin to affect bodily systems. There is a parallel model in use by critical race theorists, which states that minoritized communities experience weathering associated with chronic activation of fight-or-flight systems.^27,28^

In most scenarios in this analysis, the association among racial/ethnic composition, income inequality, and COVID-19 outcomes was positive. The only exception was the association between mortality and racial/ethnic composition when income inequality was low. This is because very few counties had low inequality and a sizable Black and Hispanic composition, yielding a greater uncertainty for estimation. The differential association between incidence and mortality and racial/ethnic composition between low and high economic inequality levels suggests that when one structural factor is at work, there is less room for the other structural factor to come into play. This association also supports the notion put forth by earlier studies for the use of composite variables that measure both income inequality and racial/ethnic composition simultaneously, such as the index of concentration at the extremes, as a critical tool for equity-focused public health surveillance.^29,30^ The overall positive associations found in this study suggest that with health equity as a stated goal of the United States, real progress on this front will likely only come with a dedicated commitment to dismantling structural racism and economic inequality, particularly racialized economic inequality.

### Limitations

This study has some limitations. First, confirmed incidence may vary by the extent of testing, and no accurate data of testing variability and accessibility exist to include in the analysis. Second, county-level associations may not be generalizable to community or neighborhood dynamics. Third, findings based on the first 200 days cannot be extended to the other durations of the COVID-19 pandemic in the United States, because the infection and mortality patterns in the initial and later periods can be different. Fourth, the study analyzed only Black and Hispanic but not other ethnic population compositions. Finally, as a cross-sectional study, the analysis does not include lagged effects or any other longitudinal means to elucidate causal pathways.

## Conclusions

This cross-sectional study reports a county-level ecological analysis of cumulative COVID-19 infection and mortality rates for the first 200 days of the pandemic in the United States since January 22, 2020, by examining the association between infection incidence and mortality on the one hand and the structural factors of racial/ethnic composition and income inequality on the other, with control for other important covariates. This analysis confirms the association between racial/ethnic composition and COVID-19 incidence and mortality. A higher level of Black or Hispanic composition in a county is associated with a higher COVID-19 incidence and mortality; a higher level of economic inequality is also associated with a higher level of incidence and mortality. The study also substantiated that the association between racial/ethnic composition and incidence/mortality is stronger when income inequality is relatively low, especially for Hispanic populations. More generally, the study suggests that high levels of income inequality may harm population health irrespective of racial/ethnic composition.

## References

[1] Adhikari S, Pantaleo NP, Feldman JM, Ogedegbe O, Thorpe L, Troxel AB Assessment of community-level disparities in coronavirus disease 2019 (COVID-19) infections and deaths in large US metropolitan areas. JAMA Netw Open. 2020;3(7):e2016938. doi:10.1001/jamanetworkopen.2020.16938 32721027 PMC7388025

[2] Millett GA, Jones AT, Benkeser D, Assessing differential impacts of COVID-19 on black communities. Ann Epidemiol. 2020;47:37-44. doi:10.1016/j.annepidem.2020.05.003 32419766 PMC7224670

[3] APM Research Lab The color of coronavirus: COVID-19 deaths by race and ethnicity in the US. Published September 10, 2020. Accessed November 12, 2020. https://www.apmresearchlab.org/covid/deaths-by-race

[4] Metzl JM, Maybank A, De Maio F Responding to the COVID-19 pandemic: the need for a structurally competent health care system. JAMA. 2020;324(3):231-232. doi:10.1001/jama.2020.9289 32496531

[5] Khazanchi R, Evans CT, Marcelin JR Racism, not race, drives inequity across the COVID-19 continuum. JAMA Netw Open. 2020;3(9):e2019933. doi:10.1001/jamanetworkopen.2020.19933 32975568

[6] Krieger N. Epidemiology and the People's Health: Theory and Context. Oxford University Press; 2011. doi:10.1093/acprof:oso/9780195383874.001.0001

[7] Wilkinson RG, Pickett K The Spirit Level: Why Greater Equality Makes Societies Stronger. Bloomsbury; 2011.

[8] Dawes D. The Political Determinants of Health. Johns Hopkins University Press; 2020.

[9] Metzl JM Dying of Whiteness. Basic Books; 2019.

[10] Carter D, May P Making sense of the US COVID-19 pandemic response: a policy regime perspective. Admin Theory Praxis. 2020;42(2):265-277. doi:10.1080/10841806.2020.1758991

[11] Kavanah N, Goel R, Venkataramani A Association of county-level socioeconomic and political characteristics with engagement in social distancing for COVID-19. Preprint. Posted online April 11, 2020. medRxiv. doi:10.1101/2020.04.06.20055632

[12] Sergent K, Stajkovic AD Women’s leadership is associated with fewer deaths during the COVID-19 crisis: quantitative and qualitative analyses of United States governors. J Appl Psychol. 2020;105(8):771-783. doi:10.1037/apl0000577 32614203

[13] Belsey T, Case A Does electoral acountability affect economic policy choices? evidence from gubernatorial term limits. Q J Econ. 1995;110(3):769-798. doi:10.2307/2946699

[14] Mahajan UV, Larkins-Pettigrew M Racial demographics and COVID-19 confirmed cases and deaths: a correlational analysis of 2886 US counties. J Public Health (Oxf). 2020;42(3):445-447. doi:10.1093/pubmed/fdaa070 32435809 PMC7313814

[15] Alsan M, Stantcheva S, Yang D, Cutler D Disparities in coronavirus 2019 reported incidence, knowledge, and behavior among US adults. JAMA Netw Open. 2020;3(6):e2012403. doi:10.1001/jamanetworkopen.2020.12403 32556260 PMC7303811

[16] Worldometer. United States coronavirus cases, deaths, recovered. Updated December 8, 2020. Accessed November 27, 2020. https://www.worldometers.info/coronavirus/country/us/

[17] Centers for Disease Control and Prevention CDC COVID data tracker: COVID-19 integrated county view. Updated December 5, 2020. Accessed November 30, 2020. https://covid.cdc.gov/covid-data-tracker/#county-view

[18] US Census Bureau. 2019 population estimates by age, sex, race and Hispanic origin. Published June 25, 2020. Accessed November 30, 2020. https://www.census.gov/newsroom/press-kits/2020/population-estimates-detailed.html

[19] US Census Bureau. ACS income inequality data tables. Published 2020 Accessed November 30, 2020. https://www.census.gov/topics/income-poverty/income-inequality/data/data-tables/acs-data-tables.html

[20] GitHub. US_County_Level_Election_Results_08-20. Published 2020 Accessed November 30, 2020. https://github.com/tonmcg/US_County_Level_Election_Results_08-20/blob/master/2016_US_County_Level_Presidential_Results.csv

[21] Kaiser Family Foundation. Status of state Medicaid expansion decisions: interactive map Published November 2, 2020. Accessed November 30, 2020. https://www.kff.org/medicaid/issue-brief/status-of-state-medicaid-expansion-decisions-interactive-map/

[22] Council of State Governments Constitutional and statutory provisions for number of consecutive terms of elected state officials. Published April 2019. Accessed November 30, 2020. http://knowledgecenter.csg.org/kc/system/files/4.9.2019.pdf

[23] National Governors Association. Governors. Published 2020 Accessed November 30, 2020. https://www.nga.org/governors/

[24] Yudell M, Roberts D, DeSalle R, Tishkoff S; 70 signatories NIH must confront the use of race in science. Science. 2020;369(6509):1313-1314. doi:10.1126/science.abd4842 32913094 PMC9994540

[25] Pickett KE, Wilkinson RG Income inequality and health: a causal review. Soc Sci Med. 2015;128:316-326. doi:10.1016/j.socscimed.2014.12.031 25577953

[26] De Maio F. Health and Social Theory. Palgrave Macmillan; 2010. doi:10.1007/978-1-137-03975-0

[27] Geronimus AT, Bound J, Waidmann TA, Rodriguez JM, Timpe B Weathering, drugs, and whack-a-mole: fundamental and proximate causes of widening educational inequity in US life expectancy by sex and race, 1990-2015. J Health Soc Behav. 2019;60(2):222-239. doi:10.1177/0022146519849932 31190569 PMC6684959

[28] Louis-Jean J, Cenat K, Njoku CV, Angelo J, Sanon D Coronavirus (COVID-19) and racial disparities: a perspective analysis. J Racial Ethn Health Disparities. 2020;7(6):1039-1045. doi:10.1007/s40615-020-00879-4 33025419 PMC7537778

[29] Krieger N, Waterman PD, Spasojevic J, Li W, Maduro G, Van Wye G Public health monitoring of privilege and deprivation with the index of concentration at the extremes. Am J Public Health. 2016;106(2):256-263. doi:10.2105/AJPH.2015.302955 26691119 PMC4815605

[30] Lange-Maia BS, De Maio F, Avery EF, Association of community-level inequities and premature mortality: Chicago, 2011-2015. J Epidemiol Community Health. 2018;72(12):1099-1103. doi:10.1136/jech-2018-210916 30171083

