# Errors in Tables 1 and 2

**DOI:** 10.1001/jamanetworkopen.2021.14136

**Published:** 2021-05-17

In the Original Investigation titled “Association of Social and Economic Inequality With Coronavirus Disease 2019 Incidence and Mortality Across US Counties,”^1^ published January 20, 2021, there was an inconsistency in the presentation of data between Tables 1 and 2. The data in Table 2 regarding population aged younger than 20 years, population aged 70 years or older, and population density should have been calculated using the same measurement units as in Table 1. Table 1 has also been amended to fix incorrect values that were given for population density. This article has been corrected.
